# Cognitive behavioral therapy for insomnia in neurodegenerative disorders—targeting sleep disturbances in Alzheimer’s and Parkinson’s disease: a scoping review

**DOI:** 10.3389/fpsyg.2025.1700496

**Published:** 2025-11-04

**Authors:** Desirèe Latella, Andrea Calderone, Carmela Casella, Rosaria De Luca, Antonio Gangemi, Federica Impellizzeri, Santina Caliri, Angelo Quartarone, Rocco Salvatore Calabrò

**Affiliations:** ^1^IRCCS Centro Neurolesi Bonino-Pulejo, Messina, Italy; ^2^Stroke Unit, AOU Policlinico G. Martino, Messina, Italy

**Keywords:** cognitive behavioral therapy for insomnia, Alzheimer’s disease, mild cognitive impairment, Parkinson’s disease, sleep disturbance, telehealth, caregiver outcomes, non-pharmacological treatment

## Abstract

**Introduction:**

Insomnia is highly prevalent in neurodegenerative disorders, yet pharmacological options carry safety and tolerability concerns. This scoping review mapped contemporary evidence for cognitive behavioral therapy for insomnia (CBT-I) across Alzheimer’s disease (AD), mild cognitive impairment (MCI), and Parkinson’s disease (PD).

**Methods:**

Following a preregistered protocol (OSF DOI: 10.17605/OSF.IO/8VP3F), we searched PubMed, Cochrane Library, Web of Science, and Scopus for studies published 2015–2025. We screened English-language studies in adults and applied dual independent review with consensus resolution. Of 105 records, 70 were screened after de-duplication, and 8 met eligibility criteria.

**Results:**

Across randomized trials, pilot and feasibility studies, and single-case experimental designs, CBT-I—delivered in person or via telehealth—consistently reduced insomnia severity and improved sleep quality, with frequent ancillary gains in mood, anxiety, and daytime functioning. Remote and digitally augmented delivery appeared feasible and acceptable for cognitively vulnerable adults and caregivers. Early signals suggested potential cognitive benefits in prodromal populations (AD/MCI), and exploratory observations linked improved sleep with plausible neurobiological mechanisms such as amyloid-beta dynamics. In PD, findings aligned with a mechanistic pathway in which presleep cognitive arousal, safety behaviors, and dysfunctional sleep beliefs are modifiable targets. Non-pharmacological comparators (e.g., mindfulness, therapeutic exercise, neuromodulation) also showed benefits, helping contextualize where CBT-I may offer disorder-relevant leverage on insomnia outcomes.

**Discussion:**

The overall strength of evidence is tempered by small samples, heterogeneity in comparators and dosing, short follow-up, and inconsistent reporting of clinically meaningful change. Priorities include multicenter randomized trials with standardized sleep and cognitive endpoints, longer observation, head-to-head comparative effectiveness with economic evaluation, adaptive protocols tailored to PD-specific disruptors, and mechanistic studies integrating digital phenotyping and biomarkers to test durability and downstream clinical impact.

## 1 Introduction

Insomnia is a common sleep disorder characterized by persistent difficulties in initiating or maintaining sleep, or early morning awakenings that impair daytime functioning and quality of life (Roth, 2007). Cognitive deficits, emotional distress, and increased healthcare utilization are all associated with chronic insomnia, which is defined as symptoms occurring at least three times per week for a period exceeding 3 months (Naha et al., 2024; Walker et al., 2022). Chronic insomnia is a global public health concern, as it affects approximately 9 to 15% of the general population (de Bergeyck and Geoffroy, 2023). Neurodegenerative diseases, such as Parkinson’s disease (PD) and Alzheimer’s disease (AD), are particularly susceptible to chronic insomnia. Beyond neurodegeneration, bipolar disorder (BD) frequently co-occurs with insomnia and anxiety. In inter-episode BD, insomnia-related sleep disruption is associated with poorer working memory and verbal learning, and improvements in sleep following CBT-I adapted for BD track with cognitive gains; importantly, anxiety is often under-measured despite its potential role as a moderator/mediator of the sleep–cognition relationship (Zhang et al., 2024). Multiple domains of health and daily functioning are significantly impaired by insomnia (Bishir et al., 2020). Sleep disturbances, including nighttime awakenings, fragmented sleep, and circadian rhythm disruption, are highly prevalent and frequently multifactorial in these conditions (Benca et al., 2022). These disturbances are the result of neurodegeneration in brain regions that regulate sleep, adverse effects of pharmacologic treatments, and coexisting psychiatric symptoms. Research indicates that persistent insomnia is experienced by 60–70% of individuals with PD and AD, which in turn exacerbates motor and cognitive symptoms, reduces quality of life, and increases caregiver burden (Aamodt et al., 2024). Deficits in attention, memory, and executive function are also exacerbated by insomnia in these populations, which disrupts daily functioning and occupational or social responsibilities (Hennawy et al., 2019; Kong et al., 2023; Medic et al., 2017). In older adults with amnestic MCI and mild AD, sleep disturbances are also linked to decrements in financial capacity, a critical instrumental ADL. In a monocentric study, caregiver-rated sleep disturbance on the Sleep Disorders Inventory predicted poorer performance on the Legal Capacity for Property Law Transactions Assessment Scale (LCPLTAS), independently of global cognition, underscoring real-world implications of insomnia for autonomy in AD/MCI (Giannouli and Tsolaki, 2023). Psychologically, it is associated with increased risks of anxiety, depression, and emotional distress (Wei et al., 2025). Conversely, prolonged sleep disruption is linked to systemic health risks, including cardiovascular disease and impaired immune function, as well as fatigue and diminished physical stamina (Onyegbule et al., 2025). Although sedative-hypnotic medications are frequently prescribed, in older adults and neurologically vulnerable populations long-term benzodiazepines and Z-drugs carry important risks (cognitive impairment, delirium, falls/fractures, tolerance/dependence), so their use is generally limited to short courses or adjunctive roles (Smichenko et al., 2020). More recently, dual orexin receptor antagonists (DORAs), suvorexant, lemborexant, daridorexant, have been approved for insomnia and show benefits on sleep onset and maintenance with favorable next-day profiles, including in cohorts ≥65 years; preliminary evidence also suggests potential utility in Alzheimer’s disease with insomnia. Against this pharmacological background, Cognitive Behavioral Therapy for Insomnia (CBT-I) remains the preferred first-line approach because it targets the behavioral and cognitive perpetuating factors of insomnia and avoids medication-related harms. Core elements are stimulus control and sleep restriction, combined with cognitive restructuring and brief sleep education/relaxation. Major clinical guidelines (AASM, ESRS, and the American College of Physicians) consistently recommend multicomponent CBT-I as first-line therapy for chronic insomnia in adults; stimulus control and sleep restriction as single-component options receive conditional recommendations, while sleep-hygiene alone is not advised as monotherapy. Typical courses comprise 4–8 sessions and can be delivered individually, in groups, or digitally (Edinger et al., 2021). Stimulus control techniques aim to re-establish a strong association between the bed and sleep by encouraging consistent sleep–wake patterns and discouraging wakeful activities in bed. Cognitive restructuring helps individuals identify and challenge negative thoughts about sleep, thereby reducing anxiety and hyperarousal (Rossman, 2019). CBT-I has demonstrated lasting efficacy in various populations and is generally well-tolerated, making it a preferable option for long-term insomnia management, particularly in vulnerable groups such as older adults or patients with neurodegenerative disorders, where pharmacological risks are elevated (Vitiello, 2016; Rossman, 2019). CBT-I has several advantages, including the absence of pharmacological side effects and sustained efficacy over time. However, challenges remain in applying it to patients with neurodegenerative disorders, who may present with cognitive limitations and reduced treatment adherence. Despite these concerns, preliminary studies suggest that CBT-I can lead to meaningful improvements in sleep quality and related symptoms, even in cognitively impaired populations, particularly when adapted (Carney et al., 2017). To date, the literature has focused primarily on AD and PD, with limited or no studies exploring the use of CBT-I in other neurodegenerative conditions. This lack of comparative data hinders a broader understanding of CBT-I’s utility across the neurodegenerative disease spectrum. This scoping review aims to synthesize current evidence on the efficacy of CBT-I in managing insomnia specifically in AD and PD, identifying existing strengths and gaps in the field, and highlighting the need for more inclusive research on other neurodegenerative disorders.

## 2 Materials and methods

**Figure 1:**
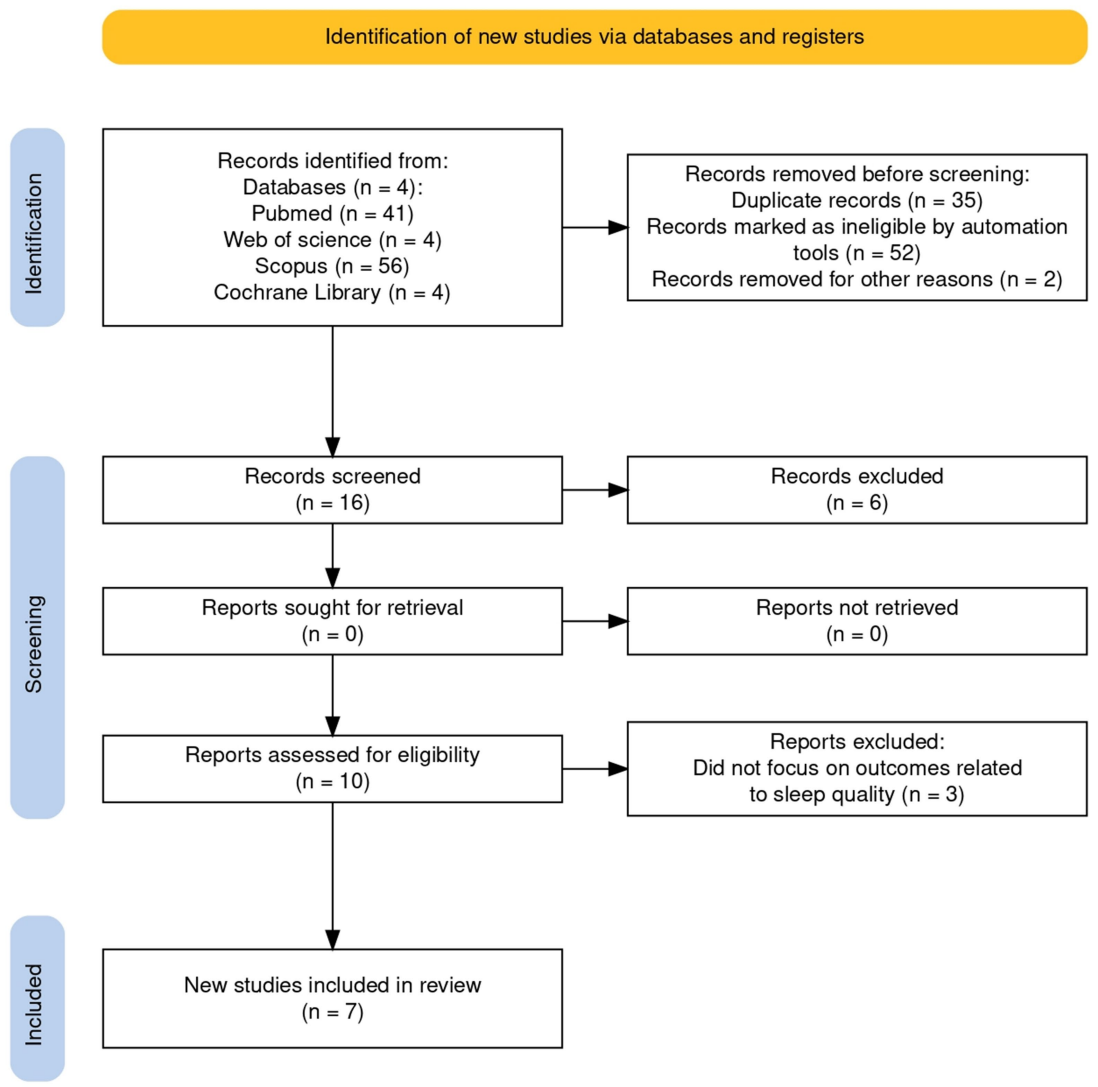
Preferred reporting items for systematic review and meta-analyses-scoping review.

This review was conducted and reported in accordance with the Preferred Reporting Items for Systematic Review and Meta-Analyses-Scoping review (PRISMA) (see Figure 1) (Haddaway et al., 2022) code/number: DOI 10.17605/OSF.IO/8VP3F, registration date: 2024-08-04.

### 2.1 Inclusion criteria

We included peer-reviewed empirical studies published in English between 2015 and 2025 that evaluated CBT-I in adults (≥18 years) with neurodegenerative disorders, with an *a priori* focus on AD, mild cognitive impairment (MCI) attributable to AD, and PD. We restricted inclusion to the past 10 years to: (i) align with contemporary DSM-5/ICSD-3 nosology that unified “insomnia disorder” (≥3 nights/week for ≥3 months), thereby reducing definitional heterogeneity; (ii) capture the post-guideline era in which multicomponent CBT-I is established as first-line and digital/group delivery became widespread; and (iii) ensure clinical relevance to current AD/PD practice. This restriction is consistent with JBI methodological guidance for scoping reviews when a justified rationale is provided, and we will transparently report this in line with PRISMA-ScR. Eligible designs comprised randomized controlled trials, quasi-experimental studies, pilot/feasibility trials, pre–post single-arm studies with adequate detail, and single-case experimental designs with replication. Studies had to deliver CBT-I in any format (individual, group, telehealth/ICBT, or blended), encompassing core elements such as stimulus control, sleep restriction, cognitive restructuring, and sleep hygiene, or clearly CBT-I informed protocols. Comparators could include wait-list, treatment-as-usual, sleep hygiene education, pharmacotherapy, or mindfulness-based programs when used as active controls.

#### 2.1.1 Insomnia definition and assessment

Insomnia was ascertained by DSM-5 or ICSD-3 diagnostic criteria (≥3 nights/week for ≥3 months with daytime impairment) or by validated threshold-based criteria on subjective scales—Insomnia Severity Index (ISI) (moderate–severe ≥15) and Pittsburgh Sleep Quality Index (PSQI) (>5)—together with frequency/duration requirements. Co-primary sleep outcomes were subjective severity (ISI, PSQI) and sleep-diary metrics (sleep onset latency [SOL], wake after sleep onset [WASO], total sleep time [TST], sleep efficiency [SE]); objective outcomes (actigraphy, polysomnography) were extracted when available. In AD/aMCI, when self-report was infeasible, we accepted caregiver/partner-proxy or assisted completion using standardized instructions; for more advanced impairment, objective or clinician-rated measures were prioritized. We prespecified subgroup analyses by ascertainment modality (diagnostic interview vs. scale threshold; self- vs. proxy-report).

#### 2.1.2 Outcomes and disease-specific measures

Beyond core sleep outcomes (ISI; PSQI; Epworth Sleepiness Scale [ESS]; Sleep Condition Indicator [SCI]; diary-derived SE/TWT/SOL/WASO; actigraphy/PSG), we extracted validated condition-tailored endpoints:

- AD/aMCI: global cognition (Montreal Cognitive Assessment [MoCA]); domain tests (e.g., RAVLT, TMT); instrumental activities (Lawton–Brody IADL; Amsterdam IADL Questionnaire [A-IADL-Q]); financial capacity (e.g., Legal Capacity for Property Law Transactions Assessment Scale [LCPLTAS]); neuropsychiatric symptoms (Neuropsychiatric Inventory [NPI]); caregiver burden (Zarit Burden Interview [ZBI]).
- PD: disease severity (Movement Disorder Society–Unified Parkinson’s Disease Rating Scale [MDS-UPDRS]); PD-specific sleep impairment (Parkinson’s Disease Sleep Scale-2 [PDSS-2]); quality of life (Parkinson’s Disease Questionnaire-8/39 [PDQ-8/PDQ-39]); cognition (MoCA; executive tasks); gait/falls/freezing of gait [FOG-Q] when reported.
- Cross-cutting mood/functional/QoL: Hospital Anxiety and Depression Scale [HADS], Beck Depression Inventory-II [BDI-II], Parkinson’s Anxiety Scale [PAS], Work and Social Adjustment Scale [WSAS], WHO Disability Assessment Schedule 2.0 [WHODAS-2], Brunnsviken Brief Quality of Life [BBQ], Stanford Self-Efficacy for Managing Chronic Disease-6 [SSES-6], EuroQoL-5D-3L [EQ-5D-3L], Parkinson’s Disease Activities of Daily Living Scale [PADLS].

To qualify, studies needed at least one validated sleep outcome (e.g., diary, actigraphy) and could also report secondary endpoints (e.g., anxiety/depression, quality of life, cognition, biomarkers). Mixed samples were eligible only if PD or AD/MCI subgroups were analyzable. For duplicate cohort reports, the most complete dataset was retained; overlapping papers were used to complement missing outcomes/timepoints. When multiple instruments assessed the same domain, we applied a predefined hierarchy and harmonized score directionality. Disease-specific measures (e.g., Montreal Cognitive Assessment Scale) were accepted. Mixed samples were eligible only when PD or AD/aMCI subgroups were analyzable. When multiple reports referred to the same cohort, the most complete dataset was retained. For overlapping reports from the same cohort, we retained the most complete dataset (largest sample/longest follow-up) and used companion papers solely to supplement missing outcomes/timepoints. When multiple instruments assessed the same domain, we applied a predefined hierarchy (disease-specific > generic; clinician-rated > self-report; continuous change > dichotomous) and harmonized score directionality.

### 2.2 Exclusion criteria

We excluded non-empirical articles (reviews, editorials, protocols, conference abstracts) and studies not published in English or outside the predefined 10-year window. Trials enrolling pediatric or adolescent populations (<18 years) or samples without a confirmed neurodegenerative diagnosis were excluded, as were caregiver-only cohorts lacking patient-level outcomes. Pharmacological-only, device-only, or exercise-only interventions were excluded unless a CBT-I arm was present. Studies were excluded if insomnia/sleep was not assessed with validated measures, if intervention details were insufficient to characterize CBT-I content/dose, or if data were inadequate for extracting pre–post or between-group effects. Multicomponent rehabilitation programs were excluded when the specific contribution of CBT-I could not be isolated. Duplicate publications and overlapping datasets were removed. Furthermore, studies with unclear diagnosis or ambiguous methods were excluded to preserve interpretability.

### 2.3 PICO evaluation

The search terms were derived using the PICO model, specifying Population, Intervention, Comparator, and Outcomes (Eriksen and Frandsen, 2018). The population we are focusing on are patients with neurodegenerative disorders specifically, AD, aMCI/prodromal and PD, who experience clinically significant insomnia. The main focus of the intervention is CBT-I, a structured, non-pharmacological treatment for insomnia, focusing on improving sleep quality and addressing the underlying causes of insomnia. To compare, we examine the traditional care or alternative treatments such as pharmacological interventions. The outcome includes improvements in in insomnia severity and sleep parameters, cognitive function, as well as cognition, psychological well-being, and overall quality of life.

### 2.4 Search strategy

Articles were selected from the following research databases: PubMed, Cochrane, Web of Science, and Scopus, using the following search terms: ((“neurodegenerative diseases”[MeSH Terms] OR (“neurodegenerative”[All Fields] AND “diseases”[All Fields]) OR “neurodegenerative diseases”[All Fields]) AND (“insomnia”[All Fields] OR “sleep initiation and maintenance disorders”[MeSH Terms] OR (“sleep”[All Fields] AND “initiation”[All Fields] AND “maintenance”[All Fields] AND “disorders”[All Fields]) OR “sleep initiation and maintenance disorders”[All Fields] OR “insomnia”[All Fields] OR “insomnias”[All Fields]) AND (“cognitive behavioral therapy”[MeSH Terms] OR (“cognitive”[All Fields] AND “behavioral”[All Fields] AND “therapy”[All Fields]) OR “cognitive behavioral therapy”[All Fields])). The search was conducted using titles and abstracts as selection criteria, and only English-language articles were included. Articles published in the last 10 years were considered, and the review was conducted by the research team across multiple databases between April and May 2025. After removing duplicates, all remaining articles were evaluated based on their title and abstract. Studies that assessed the effectiveness of CBT-I in individuals with neurodegenerative disorders were included.

### 2.5 Study selection and data extraction

A total of 105 records were retrieved from PubMed, Cochrane, Web of Science, and Scopus. After removing 35 duplicates, 70 unique records underwent screening. Of these, 17 review articles were excluded, 17 studies were removed at title screening, 14 at abstract screening, and 15 after full-text assessment, yielding 7 eligible studies on the efficacy of CBT-I in neurodegenerative disorders (AD, MCI, and PD). Screening and selection adhered to a predefined protocol and are summarized in the PRISMA flow diagram (Figure 1). Two reviewers (DL, AC) independently screened titles/abstracts and then full texts against the inclusion/exclusion criteria, with parallel, independent data extraction to limit selection and reporting biases (e.g., missing results, publication, time-lag, and language bias). Full texts deemed potentially eligible were read in full by both reviewers. Disagreements at any stage (screening or extraction) were resolved first by discussion; unresolved cases were adjudicated by a third reviewer (RSC). Inter-rater reliability was quantified using Cohen’s kappa; values >0.61 were interpreted as indicating substantial agreement, supporting the robustness of the selection and extraction procedures (McHugh, 2012). Extraction was managed in Microsoft Excel using standardized, pilot-tested templates to ensure consistency. Built-in functions (tagging, filtering, and sorting) facilitated cross-validation between reviewers, traceability of decisions, and rapid identification of discrepancies. Where multiple publications reported the same cohort, the most complete dataset was retained. Final study characteristics and outcomes were synthesized narratively and tabulated to highlight key themes in line with the review objectives (Rai et al., 2020).

## 3 Results

### 3.1 Synthesis of evidence

In the seven included studies of AD, MCI, and PD, CBT-I was implemented in different modalities (individual, group, telehealth/CBT-I); nevertheless, each of these implementations generally included the classic elements of stimulus control, sleep restriction, cognitive strategies, and sleep education. Although there was design heterogeneity (single-case experimental, pilot/feasibility, and randomized trials), the outcome measurements were largely similar, which included validated sleep measures (such as sleep diaries, and actigraphy), followed by disease-specific scales and patient-reported outcomes. The direction of effect was generally consistent: most studies found significant decreases in insomnia severity and improvements in sleep quality, and many also documented collateral gains in mood, anxiety, daily living skills, or quality of life. Digital delivery (including telehealth and app-driven CBT-I) proved to be feasible and acceptable in older and/or cognitively impaired subjects in general, with high adherence. Signals of cognitive benefit were early (more apparent in MCI and at-risk populations), and biomarker results (where studied) were exploratory. The duration of effect has been shown in some studies of short-term follow-up, but sustained effects and their effects on disease progression were rarely investigated. Comparator arms included wait-list and sleep hygiene education, as well as other non-pharmacological therapies, supporting the importance of head-to-head trials. In summary, we found the evidence supporting the feasibility of, and potential efficacy for, CBT-I in sleep outcomes across AD/MCI and PD, suggesting a need for large, long, and standardized assessment in the context of small sample, variable dosing, and limited follow-up as critical limitations. We next detail these findings by condition, first reviewing evidence for CBT-I in PD, then in AD and MCI.

### 3.2 The effectiveness of CBT-I in Parkinson’s disease

Insomnia is a common and debilitating symptom in PD, affecting approximately 60–70% of patients. Sleep disturbances can exacerbate both motor and non-motor symptoms, such as depression, anxiety, and cognitive dysfunction, making effective treatment crucial for improving overall quality of life. In this context, CBT-I has shown promise as a non-pharmacological treatment for managing insomnia in PD patients. Lebrun et al. (2020) conducted a multiple-baseline single-case experimental design to assess the effectiveness of CBT-I in individuals with insomnia disorder (ID) comorbid to PD. This study found that CBT-I led to significant improvements in sleep quality, reduced insomnia severity, and positive changes in daytime functioning. Notably, CBT-I also resulted in improvements in psychological functioning, including mood and anxiety symptoms, which are often affected in PD patients. These improvements were maintained at a 3-month follow-up, suggesting that CBT-I could provide lasting benefits for PD patients dealing with insomnia (Lebrun et al., 2020). In a complementary observational study, Lebrun et al. (2019) examined 68 individuals without dementia with idiopathic PD and tested a serial mediation model linking presleep cognitive arousal to ID via sleep-related safety behaviors and strongly endorsed dysfunctional sleep beliefs.

The association between presleep cognitive arousal and ID was explained sequentially by greater engagement in sleep-related safety behaviors and stronger endorsement of dysfunctional sleep beliefs, whereas the alternative ordering (beliefs preceding safety behaviors) was not supported.

This pattern aligns closely with CBT-I’s theoretical targets, highlighting safety behaviors and maladaptive cognitions as proximal, modifiable drivers of persistent insomnia in PD. By mapping these pathways with validated self-report measures, the study offers mechanistic context for the symptomatic gains observed with CBT-I and suggests why benefits may extend to daytime functioning (Lebrun et al., 2019). Similarly, Kraepelien et al. (2020) conducted a randomized controlled trial (RCT) to evaluate the effects of internet-based CBT-I (ICBT) for PD patients. Participants were randomized to receive either ICBT combined with standard medical treatment or to a waitlist control group. The results showed that ICBT significantly improved daily functioning, insomnia symptoms, anxiety, and depression compared to the control group. However, only about one-third of participants in the treatment group were classified as treatment responders, indicating that the treatment might need further adjustments to enhance its efficacy for all PD patients.

These findings highlight that while CBT-I is effective in improving sleep and related symptoms, individual responses vary, and further research is needed to refine treatment protocols (Kraepelien et al., 2020). However, further studies focusing specifically on CBT-I in PD are needed to compare the efficacy of these approaches directly. These studies indicate that CBT-I is an effective and feasible treatment for managing insomnia in PD, improving both sleep quality and psychological well-being. Nevertheless, the variation in treatment response and the need for further optimization of CBT-I protocols in PD patients highlight the importance of personalizing treatments to maximize benefits. Future research should focus on long-term outcomes, including motor function and cognitive performance, to assess whether improving sleep through CBT-I can help mitigate other PD-related symptoms and slow disease progression. See Table 1 for a detailed overview.

**Table 1:** Summary of PD studies included in the research.

Author	Aim of the study	Study design	Intervention	Sample size	Outcome measures	Main findings
Lebrun et al. (2020)	The first aim is to determine the effect of CBT-I on both nighttime and daytime insomnia related symptoms in ID comorbid to PD. The second aim is to evaluate the impact of CBT-i on various mental health indicators in ID comorbid to PD.	Multiple-baseline AB single-case experimental design SCED across subjects	CBI-i treatment consisted of 8 weekly, individual-administered therapy sessions of approximately 45–90 min, combining behavioral, cognitive, and educational strategies	15 participants with insomnia disorder comorbid to PD	Disease severity was assessed with the Movement Disorders Society-UPDRS; Disease progression was assessed by the modified Hoehn and Yahr scale. Current ID, mood, and anxiety disorders were diagnosed as per the ICSD-3 criteria; The Expanded Consensus Sleep Diary for evening (CSD-E) was completed thorough the study phases (baseline and follow-ups); Sleep condition indicator (SCI); Insomnia severity index (ISI); Sleep-related behaviors questionnaire 20 (SRBQ-20); Short version of the dysfunctional beliefs and attitudes about sleep (DBAS-16); Presleep arousal scale (PSAS); Self-efficacy for sleep scale (SES); Beck depression inventory (BDI-II); Parkinsonʼs anxiety scale (PAS); Quality of life, 3-level version of EQ-5D questionnaire; Treatment acceptability questionnaire (TAQ)	CBT-i was associated with significant changes in sleep variables and ID criteria. Significant positive treatment related effects were also noted for all indices of daytime and psychological functioning, and for variables perpetuating ID. All these improvements were well maintained at 3-month follow-up.
Lebrun et al. (2019)	The aim is to examine a serial meditation model of sleep-related safety behaviors and dysfunctional beliefs about sleep in association with pre sleep cognitive arousal and insomnia disorder in patients with PD	Observational study	Not Specificated.	68 nonconsecutive individuals without dementia with idiopathic PD	Motor experiences of daily living and motor disability were assessed using, respectively, Part II of the Movement Disorder Society of the Unified Parkinson’s Disease Rating Scale (MDS-UPDRS) and the modified Hoehn and Yahr staging. Self-report measures included the French version of the Sleep Condition Indicator, the Sleep-Related Behaviors Questionnaire (SRBQ-20), the Dysfunctional Beliefs and Attitudes about Sleep (DBAS-16), the cognitive subscale of the Presleep Arousal Scale (PSAS-C), the Beck Depression Inventory-II (BDI-II) and the Parkinson’s Anxiety Scale (PAS)	The association between presleep cognitive arousal (PSAS-C) and insomnia disorder was serially mediated by sleep-related safety behaviors (SRBQ-20) and strong endorsement of dysfunctional beliefs about sleep (DBAS-16). An alternate serial mediation model in which dysfunctional beliefs about sleep precede sleep-related safety behaviors was not statistically significant.
Kraepelien et al. (2020)	The aim of the study is to evaluate the effects of an ICBT-program for people with Parkinson on daily functioning, in comparison to being on a waitlist to ICBT (internet-based cognitive behavioral therapy), as well as to further explore involvement, treatment satisfaction and patients’ subjective evaluations. The aim was also to investigate the effects on secondary outcome measures such as depressive symptoms, anxiety, insomnia symptoms, and quality of life.	Randomized Controlled Trial.	Patients were randomized to 10 weeks of either ICBT combined with standard medical treatment, or standard medical treatment plus being on waitlist to ICBT.	The study involved 77 individuals with PD.	WSAS (Work and Social Adjustment Scale); Hospital Anxiety and Depression Scale (HADS-A) and depression (HADS-D); the Insomnia Severity Index (ISI); the Parkinson’s Disease Questionnaire-8, the World Health Organization Disability Assessment Schedule 2-12-item (WHODAS-2); the Brunnsviken Brief Quality of life scale (BBQ); the Stanford Self-Efficacy for Managing Chronic Disease (SSES6); Clinical Global Impression-Severity and Improvement scales (CGI-S, CGI-I)	Participants receiving ICBT reported significantly higher functioning after treatment (WSAS group difference–4.56, controlled effect size *g* = 0.69, significant group by time interaction, Wχ^2^ = 26.23, *p* = 0.001). However, only around one third of participants in the treatment group were classified as treatment responders, defined as having a 30% reduction on the WSAS post treatment. Patient involvement and ratings of ICBT credibility were high. Symptoms of anxiety, depression and insomnia symptoms were significantly lower after treatment compared to control group. There were also positive effects on Parkinson-specific function and quality of life in the treatment group.

### 3.3 The effectiveness of CBT-I in AD and MCI

In five studies spanning AD, MCI, and caregiver contexts, sleep outcomes consistently favored behavioral interventions, with CBT-I showing the most uniform effects on validated insomnia metrics and emerging signals on cognition. Among AD caregivers, a micro-costing comparison reported that mindfulness-based MAP-I achieved sleep improvements comparable to CBT-I while being less resource-intensive, indicating that, in caregiving populations, scalable behavioral options can yield similar symptomatic gains with different cost profiles (Bentley et al., 2022). In a telehealth randomized trial enrolling older adults with insomnia complaints, participants were assigned to standard CBT-I, digitally augmented CBT-I (dashboard, smartphone app, connected devices), or sleep hygiene education over six weekly sessions; available materials emphasize feasibility, engagement, and an *a priori* focus on ISI and actigraphy, supporting the acceptability of remote CBT-I in cognitively vulnerable groups without over-interpreting yet-to-be-reported endpoints (Emert et al., 2023). Two patient-focused studies provide preliminary clinical signals relevant to AD risk and prodromal disease. In a single-site pilot RCT of older adults with insomnia symptoms and AD risk, an eight-session CBT-I course produced reductions in insomnia severity and improvements in sleep quality, with exploratory indications that better sleep might relate to attenuated Aβ accumulation, hypothesis-generating findings that frame plausible mechanistic pathways rather than definitive disease modification (Siengsukon et al., 2020). In individuals with MCI, an empowerment-based adaptation of CBT-I was associated with meaningful improvements in ISI alongside concurrent gains in cognitive performance, suggesting that targeting insomnia at prodrome may deliver dual symptomatic and cognitive benefits (Li and Yu, 2024). Finally, in a small dementia cohort, therapeutic exercise and NESA neuromodulation outperformed usual care on Pittsburgh Sleep Quality Index (PSQI) at 5–7 months, with better daytime sleepiness in the neuromodulation arm and cognitive improvements observed in both active groups while controls worsened; although not CBT-I, these comparators underscore that non-pharmacological approaches can shift sleep and cognition trajectories in neurodegenerative disease, contextualizing the behavioral ecosystem in which CBT-I operates (Teruel-Hernández et al., 2023).

Taken together, these results point to consistent improvements in insomnia severity and sleep quality with CBT-I across AD-relevant populations, feasible remote delivery, and early signals for cognitive benefit in at-risk or MCI cohorts. At the same time, variability in comparators and dosing, small samples, and limited long-term follow-up temper conclusions about durability and downstream neurodegenerative outcomes, motivating larger trials with standardized sleep and cognitive endpoints (Bentley et al., 2022; Emert et al., 2023; Siengsukon et al., 2020; Li and Yu, 2024; Teruel-Hernández et al., 2023). See Table 2 for a detailed overview.

**Table 2:** Summary of AD/MCI studies included in the research.

Author/diagnosis	Aim of the study	Study design	Intervention	Sample size	Outcome measures	Main findings
Emert et al. (2023) Diagnosis: (AD)	The aims are to evaluate the difference between the active treatment comparator and the active control comparator on insomnia severity and on sleep metrics. The exploratory aim is to compare active treatments to active control comparator on cognitive performance, therapeutic adherence and neurodegenerative and systemic inflammatory blood-based biomarkers.	Randomized Controlled Trial.	All participants were randomly assigned to one of three telehealth interventions delivered through 6-weekly sessions: (1) CBTi augmented with a clinician-patient dashboard, smartphone application, and integrated smart devices; (2) standard CBTi; (3) sleep hygiene education	100 participants with complaints of insomnia	Self-report assessment measures were completed by participants on REDCap; MoCA; ISI; Consensus Sleep Diary; Dysfunctional Beliefs and Attitudes about Sleep; Sleep Condition Indicator; Sleep Need Questionnaire; Glasgow Sleep Effort Scale; PROMIS Sleep Related Impairment; Multidimensional Fatigue Index. Circadian Measures: Actigraphy (Actiwatch Spectrum); Apple Watch. Biomarkers: Optional blood draw Cognitive: Mobile Monitoring of Cognitive Change. Health Behaviors: Oxygen Monitor; Self-reported Height & Weigh; HANDLS Physical Activity; HANDLS Physical Health Functioning; PROMIS Pain Intensity & Interference; PROMIS Global Health Questionnaire; Patient Health Questionnaire.	The study protocol demonstrates efficacy and feasibility of this approach with an intensive older adult therapeutic course. For the primary outcome of self-reported ISI, a meta-analysis comparing CBTi to SHE has reported a between group effect size (Hedge’s g) of 0.92.61 For either our CBTi our CBTi+Sleep Space conditions, 36 experimental condition participants and 18 SHE participants yields 88% power to detect an effect size of 0.92 or greater. For the comparison between CBTi and CBTi+Sleep Space conditions, while the influence of Sleep Space is not known *a priori*, a comparison of two conditions with 36 participants each has 80% power to detect an effect as small as *d* = 0.67.
					Psychosocial Measures: Perceived Stress Scale; Perceived Stress Questionnaire, Generalized Anxiety Disorder; Geriatric Depression Scale; Life Aging Satisfaction; Subjective Age; HANDLS Fall History; Medication or Therapy Changes; Everyday Discrimination Scale Vigilant Coping; Neighborhood Disorder Cohesion; Hawthorne Questionnaire; Tech User Questions	
Teruel-Hernández et al. (2023) Diagnosis: (Dementia)	The aim of this study was to test the effect of TE and non-invasive neuromodulation through the NESA device (NN) on sleep quality, daytime sleepiness, and cognitive function	Randomized Multicenter Clinical Trial	In the Therapeutic Exercise protocol, the participants received 52 sessions of an adapted cardiovascular exercise program. In the NESA protocol the participants underwent 20 sessions of the non-invasive neuromodulation technique.	30 patients diagnosed with dementia were randomized as follow: 10 participants were assigned to the NESA protocol (NNG), 10 to the Therapeutic Exercise Protocol (TEG) and 10 to the control group (CG)	Sleep quality was evaluated using the PSQI; Daytime sleepiness was evaluated using the ESS; Cognitive function was evaluated by means of the Mini-Cognitive Examination Test (Lobo’s MEC)	Comparing the three groups using the PSQI, significant differences were found after 5 months (*p* = 0.048) and after 7 months (*p* = 0.002). Both the NNG and TEG groups showed improvements in sleep quality after 7 months, while the CG showed worsening sleep quality. The NNG group exhibited better daytime sleepiness outcomes compared to the CG, which worsened. Significant improvements in cognitive function were observed in both the NNG (score of 30.7) and TEG (score of 27.5) groups, while the CG showed a small decline.
Siengsukon et al. (2020)	This study explores how CBT-I may improve cognitive function in patients with insomnia symptoms and a potential risk for AD, suggesting CBT-I may slow the accumulation of Aβ, a biomarker associated with AD.	Randomized Controlled Trial	CBT-I therapy consisting of 8 weekly sessions, combining cognitive, behavioral, and educational strategies	60 participants with insomnia and cognitive concerns related to AD risk	ISI, Cognitive Function tests, Amyloid-β deposition measurement	CBT-I was associated with significant improvements in sleep quality and reduction in insomnia severity, with evidence suggesting a possible impact on amyloid-β accumulation, which may be relevant for AD.
Diagnosis: (AD)						
Li and Yu (2024) Diagnosis: (MCI)	This pilot study examined the use of empowerment-based CBT-I in patients with MCI, showing significant improvements in sleep and cognitive function, which is relevant for AD as MCI is often a preclinical phase of dementia.	Pilot Study	Empowerment-based CBT-I intervention adapted for individuals with MCI	40 participants with MCI	ISI, Cognitive performance tests, Sleep quality measures	Significant improvements in sleep quality and cognitive function in MCI patients, with promising implications for slowing the progression to AD.

Alzheimer’s disease (AD); Mild cognitive impairment (MCI); Cognitive Behavioral Therapy for Insomnia (CBT-I); Sleep Hygiene Education (SHE); amyloid-β (Aβ); Montreal Cognitive Assessment (MoCA); Insomnia Severity Index (ISI); Pittsburgh Sleep Quality Index (PSQI); Epworth Sleepiness Scale (ESS); Mini-Cognitive Examination Test (MEC); Patient-Reported Outcomes Measurement Information System (PROMIS); Healthy Aging in Neighborhoods of Diversity across the Life Span (HANDLS); Research Electronic Data Capture (REDCap); Therapeutic Exercise (TE); NESA neuromodulation (NESA); NESA neuromodulation (NN); NESA Neuromodulation Group (NNG); Therapeutic Exercise Group (TEG); Control Group (CG).

## 4 Discussion

### 4.1 Qualitative synthesis and contribution of this scoping review

This scoping review consolidates a fragmented evidence base on CBT-I across neurodegenerative conditions by juxtaposing findings in AD, MCI, and PD. The novelty of our work lies in mapping not only clinical signals on validated sleep outcomes but also the delivery formats (in-person vs. telehealth/ICBT), adjacent behavioral comparators (e.g., mindfulness, therapeutic exercise, neuromodulation), and early mechanistic readouts (e.g., exploratory amyloid-β (Aβ) trajectories in at-risk older adults). Rather than estimating pooled effects, we delineate patterns that persist despite heterogeneity in design, single-case experimental, pilot/feasibility, and randomized controlled trials, and outcome selection. Several consistent, practice-relevant messages emerge. First, CBT-I is feasible and acceptable in cognitively vulnerable populations, including when delivered remotely, with good engagement reported where documented (Emert et al., 2023; Kraepelien et al., 2020). Second, across AD/MCI and PD, most studies describe reductions in insomnia severity and improved sleep quality, with frequent ancillary gains in mood, anxiety, or daily functioning (Lebrun et al., 2020; Li and Yu, 2024; Siengsukon et al., 2020). Third, selected reports suggest cognitive signals in MCI or at-risk cohorts and plausible mechanistic pathways, for example, links between presleep cognitive arousal, safety behaviors, and dysfunctional beliefs (Lebrun et al., 2019), or preliminary indications that improved sleep may relate to attenuated Aβ accumulation (Siengsukon et al., 2020). Finally, by situating CBT-I alongside other non-pharmacological approaches (mindfulness, exercise, neuromodulation), this review clarifies the behavioral ecosystem in which CBT-I operates, highlighting both the comparative strengths of CBT-I for insomnia endpoints and the potential complementarity of alternative modalities (Bentley et al., 2022; Teruel-Hernández et al., 2023). For clarity, we reference non–CBT-I behavioral approaches (e.g., mindfulness) here as contextual evidence but did not include them in the Results synthesis following eligibility refinement. Together, these observations extend the field’s understanding from isolated trials to a condition-stratified, method-aware synthesis that can guide future program design and evaluation in neurodegenerative care. In the following sections, we critically appraise the evidence, organizing the discussion thematically: first addressing studies on AD and MCI, followed by findings pertaining to PD, in order to delineate condition-specific outcomes and therapeutic implications.

### 4.2 CBT-I in AD and MCI: sleep gains, early cognitive signals, and feasibility

In AD-relevant settings, five studies collectively point toward beneficial sleep outcomes with CBT-I and allied behavioral interventions, while also emphasizing feasibility in older adults and caregivers. Among caregivers, a micro-costing comparison indicated that mindfulness-based Mindful Awareness Practices for Insomnia (MAP-I) achieved sleep improvements comparable to CBT-I at lower cost, suggesting that resource-sensitive behavioral options can yield similar symptomatic gains in this subgroup (Bentley et al., 2022). Although not designed to adjudicate mechanistic effects, these findings imply that caregiver-targeted behavioral interventions may mitigate insomnia burden without compromising efficiency, an observation of practical import for services planning. A telehealth randomized trial in older adults assigned participants to standard CBT-I, digitally augmented CBT-I (dashboard, smartphone app, connected devices), or sleep hygiene education over six weekly sessions. While publicly available reports emphasize feasibility, adherence, and power estimates rather than final endpoints, the trial’s structure targets reductions in insomnia severity and the inclusion of actigraphy reflects a rigorous approach to sleep measurement in a remote format (Emert et al., 2023; Ritterband et al., 2025; Espie et al., 2019). The key contribution here is implementation-focused: it demonstrates that remote CBT-I delivery is acceptable and operationally viable in cognitively vulnerable older adults, an increasingly relevant consideration for health systems. Two patient-focused studies provide early clinical signals at the interface of sleep and cognition. In a single-site pilot RCT of older adults with insomnia symptoms and risk for AD, an eight-session CBT-I course produced reductions in insomnia severity and better sleep quality, accompanied by exploratory indications that improved sleep might relate to attenuated Aβ accumulation (Siengsukon et al., 2020; Shokri-Kojori et al., 2018; Lucey, 2020). While preliminary and hypothesis-generating, this pattern aligns with the proposition that sleep consolidation may modulate neurobiological processes relevant to AD risk. In participants with MCI, an empowerment-based adaptation of CBT-I yielded meaningful improvements on sleep metrics (e.g., ISI) alongside concurrent gains in cognitive performance (Li and Yu, 2024; Zhang et al., 2022; Ungvari et al., 2025; Lacerda et al., 2024). The co-movement of sleep and cognition suggests CBT-I may confer dual benefits at prodromal stages, though confirmatory multi-site trials with standardized neurocognitive batteries and longer follow-up are needed. Finally, within a small dementia cohort, therapeutic exercise and NESA neuromodulation outperformed usual care on the PSQI at 5–7 months, with daytime sleepiness improving in the neuromodulation arm and cognitive scores rising in both active arms while the control group worsened (Teruel-Hernández et al., 2023). Although these comparators are not CBT-I, their favorable trajectories underscore the plasticity of sleep and cognition in neurodegenerative disease and contextualize where CBT-I sits among other non-pharmacological strategies. Taken together, the AD/MCI evidence supports CBT-I as clinically promising for sleep outcomes with feasible remote delivery, and it surfaces early cognitive signals in prodromal or at-risk groups. The novelty relative to prior literature lies less in isolated efficacy claims and more in the convergent pattern across modalities, settings, and measures that collectively motivates larger, more harmonized trials (Furukawa et al., 2024).

### 4.3 Mapping the PD insomnia pathway: rationale and effects of CBT-I

The PD literature similarly supports CBT-I’s utility for sleep and psychological outcomes while offering mechanistic insight. In a multiple-baseline single-case experimental design, CBT-I produced robust improvements in sleep variables and ID criteria, with daytime and psychological functioning also improving and gains maintained at 3 months (Lebrun et al., 2020). These patient-level trajectories are instructive in PD, where heterogeneity in motor and non-motor symptoms can obscure average effects (Duan et al., 2025; Iranzo et al., 2024; Kalmbach et al., 2020). Complementing these findings, an observational study tested a serial mediation model and showed that the link between presleep cognitive arousal and ID was sequentially mediated by sleep-related safety behaviors and dysfunctional sleep beliefs; the reverse ordering was unsupported (Lebrun et al., 2019). This pathway aligns closely with CBT-I’s targets, reinforcing the therapeutic rationale in PD by specifying modifiable drivers of persistent insomnia.

A randomized controlled trial of individually tailored ICBT reported better daily functioning and lower anxiety, depression, and insomnia symptoms versus wait-list, albeit with about one-third of participants meeting treatment-responder criteria (Kraepelien et al., 2020).

The mixed response rate highlights a recurring theme in PD: even with evidence-based content, heterogeneous clinical profiles (e.g., nocturia, motor fluctuations, medication timing) likely require stratified or adaptive protocols to optimize benefit. Finally, a videoconference-delivered mindfulness-based intervention showed modest improvements across several symptoms, including insomnia, relative to wait-list (Bogosian et al., 2022). Although mindfulness is not CBT-I, the signal adds context, suggesting that behavioral self-management more broadly can support sleep and well-being in PD, with CBT-I offering a more insomnia-specific lever. Collectively, PD studies converge on a favorable direction of effect for sleep and mood outcomes with CBT-I, provide a mechanistic scaffold (cognitive arousal, safety behaviors, dysfunctional beliefs), and spotlight response heterogeneity that likely hinges on PD-specific sleep disruptors. The novel contribution of this synthesis is to integrate design-level evidence (RCT) with mechanism-focused observations, thereby clarifying not only that CBT-I helps many PD patients but also why and for whom optimization is needed.

### 4.4 Strengths, limitations, and future directions

A key strength of this review is its condition-stratified synthesis that spans AD/MCI and PD, integrates multiple delivery models (in-person, ICBT/telehealth, digitally augmented), and attends to mechanistic and pragmatic dimensions (e.g., caregiver cost profiles, feasibility, adherence). We emphasized validated sleep measures (e.g., PSQI, sleep diaries, actigraphy) and, where available, disease-specific or cognitive endpoints to preserve clinical interpretability. Another strength is the method awareness across designs, which allows readers to weigh signals emanating from pilot/feasibility work against those from controlled trials, and to appreciate the added explanatory value of single-case methodologies in heterogeneous populations like PD. Limitations reflect the underlying literature. Several studies are small or single-site, often with short follow-up, which constrains inferences about durability and downstream clinical impact (e.g., cognitive decline trajectories in AD/MCI or motor outcomes in PD). Reporting of minimal clinically important differences and responder definitions is inconsistent across studies, complicating judgments about clinical meaningfulness beyond statistical significance. The heterogeneity of comparators (sleep hygiene, wait-list, mindfulness, exercise, neuromodulation) precludes straightforward cross-study contrasts and obscures relative effectiveness. In AD/MCI, promising mechanistic signals, such as potential links between improved sleep and Aβ dynamics, remain exploratory, not definitive (Siengsukon et al., 2020). In PD, response variability suggests that unmeasured moderators (nocturia, dopaminergic schedules, REM behavior disorder, pain, autonomic dysregulation) may dilute group-level effects and warrant tailored algorithms. Finally, remote-delivery studies underscore feasibility but often prioritize process metrics over fully reported clinical endpoints (Emert et al., 2023), limiting quantitative synthesis. Looking ahead, we see several priorities. First, larger, multi-site randomized trials with longer follow-up are needed to clarify the maintenance of sleep gains and test clinically meaningful thresholds on standardized measures across diseases. Second, head-to-head comparisons between CBT-I and alternative non-pharmacological strategies (e.g., mindfulness, exercise, neuromodulation) should be powered to detect non-inferiority/superiority on sleep and second-order outcomes (mood, function, caregiver burden), with economic evaluations embedded (Bentley et al., 2022). Third, mechanistic studies should integrate digital phenotyping (actigraphy, wearables), biomarkers (e.g., Aβ in at-risk cohorts), and cognitive batteries to test whether sleep consolidation yields measurable changes in neurocognitive trajectories. Fourth, in PD, adaptive or stratified CBT-I protocols that explicitly address disease-specific sleep disruptors (e.g., nocturia management strategies, medication-timing psychoeducation) may increase responder rates; implementation studies should examine who benefits most and under what delivery conditions. Finally, across conditions, telehealth and digitally augmented CBT-I warrant further testing of engagement, equity, and scalability, including accessibility for patients with cognitive impairment and support for caregivers who often co-manage treatment routines.

### 4.5 Language and cultural considerations

Most included studies were conducted in English and in Western settings, which may introduce language/cultural bias. Although meta-epidemiological analyses in conventional medicine often find minimal impact of English-only restrictions on pooled effect estimates, sleep and neurocognitive outcomes are culture-sensitive (e.g., norms about bed-partner presence, napping, household structure) Widely used instruments, including the PSQI and ISI, have multiple cross-cultural validations supporting broader applicability; however, measurement invariance cannot be assumed *a priori*. Future updates should include non-English databases, extract language/setting as moderators, and, where possible, test invariance across translations to better assess generalizability.

### 4.6 Social isolation, loneliness, and non-specific effects

Evidence links loneliness/social isolation with poorer sleep quality in older adults. Accordingly, part of the apparent benefit of behavioral interventions, including CBT-I, especially when group-based or supported by regular human contact, may reflect non-specific social effects (reduced isolation, increased perceived support) in addition to the therapy’s specific mechanisms (stimulus control, sleep restriction, cognitive restructuring). This possibility does not detract from CBT-I’s therapeutic value but suggests that delivery modality (group vs. individual vs. digital), frequency of contact, and baseline isolation/loneliness may moderate outcomes. Future trials should (i) measure loneliness/social isolation alongside sleep endpoints, (ii) prespecify analyses by delivery format and human-contact intensity, and (iii) consider adjuncts that address social connectedness for highly isolated older adults.

## 5 Conclusion

This scoping review maps contemporary evidence for CBT-I in neurodegenerative contexts, centering on AD, MCI, and PD. Across heterogeneous designs, CBT-I consistently reduced insomnia severity and improved sleep quality. In PD, benefits extended to mood and daytime functioning, supported by mechanistic links between cognitive arousal, safety behaviors, and beliefs. In AD/MCI and at-risk cohorts, early signals suggest potential cognitive gains and plausible biomarker relevance, though preliminary. Remote and digitally augmented delivery proved feasible, expanding access for cognitively vulnerable adults and caregivers. Nonetheless, small samples, short follow-up, and variable comparators limit inferences about durability and disease modification. Response heterogeneity in PD highlights the need for tailored protocols that account for motor fluctuations, nocturia, and medication timing. In AD/MCI, harmonized cognitive endpoints and longer observation are required to test clinically meaningful change. Comparative effectiveness studies should position CBT-I alongside mindfulness, exercise, and neuromodulation with embedded economic evaluation. Implementation work must address equity, caregiver involvement, and real-world adherence in telehealth pathways. Mechanistic trials integrating wearables and fluid biomarkers could clarify how sleep consolidation translates to neurocognitive outcomes. Collectively, CBT-I is a pragmatic, scalable candidate for routine care, warranting definitive multicenter RCTs to secure durable, patient-relevant benefits.

## Data Availability

The original contributions presented in the study are included in the article/supplementary material, further inquiries can be directed to the corresponding author.
